# Improving the safety of cell therapy products by suicide gene transfer

**DOI:** 10.3389/fphar.2014.00254

**Published:** 2014-11-27

**Authors:** Benjamin S. Jones, Lawrence S. Lamb, Frederick Goldman, Antonio Di Stasi

**Affiliations:** ^1^Bone Marrow Transplantation and Cellular Therapy Unit, Division of Hematology-Oncology, Department of Medicine, The University of Alabama at BirminghamBirmingham, AL, USA; ^2^Division of Hematology Oncology, Department of Pediatrics, The University of Alabama at BirminghamBirmingham, AL, USA

**Keywords:** suicide gene, cell therapy, iCasp9, CAR T cells, TCR redirected T cells, HSV-tk

## Abstract

Adoptive T-cell therapy can involve donor lymphocyte infusion after allogeneic hematopoietic stem cell transplantation, the administration of tumor infiltrating lymphocyte expanded *ex-vivo*, or more recently the use of T cell receptor or chimeric antigen receptor redirected T cells. However, cellular therapies can pose significant risks, including graft-vs.-host-disease and other on and off-target effects, and therefore strategies need to be implemented to permanently reverse any sign of toxicity. A suicide gene is a genetically encoded molecule that allows selective destruction of adoptively transferred cells. Suicide gene addition to cellular therapeutic products can lead to selective ablation of gene-modified cells, preventing collateral damage to contiguous cells and/or tissues. The “ideal” suicide gene would ensure the safety of gene modified cellular applications by granting irreversible elimination of “all” and “only” the cells responsible for the unwanted toxicity. This review presents the suicide gene safety systems reported to date, with a focus on the state-of-the-art and potential applications regarding two of the most extensively validated suicide genes, including the clinical setting: herpes-simplex-thymidine-kinase and inducible-caspase-9.

## Introduction

Adoptive T-cell therapy can involve donor lymphocyte infusion (DLI) after allogeneic hematopoietic stem cell transplantation (allo-HSCT) (Copelan, 2006; Bar et al., 2013), the administration of tumor infiltrating lymphocyte (TILs) expanded *ex-vivo* (Dudley et al., 2013), or more recently the use of T cell receptor (TCR) or chimeric antigen receptor (CAR) redirected T cells (Cieri et al., 2014).

Cellular therapies are not without risks. Donor T cells within the HSC product or infused post-transplant as DLI have been associated with potentially fatal graft-vs.-host-disease (GVHD) (Copelan, 2006), whereas administration of engineered T cells has also resulted in on/off target toxicities as well as a cytokine release syndrome (Tey, 2014).

Unlike small molecules or biologics, cell therapies have a very long, or even an indefinite half-life, therefore since toxicity can be progressive a safety switch is needed in order to eliminate the infused cells in case of adverse events. A suicide gene is a genetically encoded molecule that allows selective destruction of adoptively transferred cells. Suicide gene addition to cellular therapeutic products can lead to selective ablation of gene-modified cells, preventing collateral damage to contiguous cells and/or tissues. The “ideal” suicide gene would ensure the safety of gene modified cellular applications by granting irreversible elimination of “all” and “only” the cells responsible for the unwanted toxicity.

This review presents the suicide gene safety systems reported to date, with a focus on the state-of-the-art and potential applications regarding two of the more extensively validated suicide genes in the clinical setting: herpes-simplex-thymidine-kinase (HSV-TK) and inducible-caspase-9 (iCasp9).

## Available suicide gene technologies

Suicide gene technologies can be broadly classified based upon their mechanism of action in metabolic (gene-directed enzyme prodrug therapy, GDEPT), dimerization inducing, and therapeutic monoclonal antibody mediated. The ideal agent for suicide gene activation should be biologically inert, have an adequate bio-availability and bio-distribution profiles, and be characterized by intrinsic acceptable or absent toxicity.

GDEPT (Springer and Niculescu-Duvaz, 2000) converts a nontoxic drug to a toxic compound in gene-modified cells. Examples include herpes simplex virus thymidine kinase (HSV-TK) (Ciceri et al., 2007, 2009), and cytosine deaminase (CD) (Tiraby et al., 1998). Unlike the mammalian thymidine kinase, HSV-TK is characterized by 1000 fold higher affinity to specific nucleoside analogs (Elion et al., 1977), including ganciclovir (GCV), making it suitable for use as a suicide gene in mammalian cells. Mechanistically, HSV-TK phosphorylates nucleoside analogs, including acyclovir and GCV, and their resulting triphosphate form incorporates into DNA via the action of DNA polymerase, leading to chain termination and cell death (Moolten, 1986). HSV-TK/GCV also induces apoptosis through CD95-L independent CD95 aggregation, leading to the formation of a Fas-associated death domain protein (FADD) and caspase-8-containing death-inducing signaling complex (Beltinger et al., 1999). Similarly, the CD gene encodes cytosine deaminase, which converts 5-fluorocytosine (5-FC) into the cytotoxic 5-fluorouracil (5-FU) (Tiraby et al., 1998).

Apoptotic genes (e.g., C*aspases*) eliminate cells by inducing apoptosis (Yamabe et al., 1999; Carlotti et al., 2005; Di Stasi et al., 2011; Zhou et al., 2014). Chimeric proteins composed of a drug binding domain linked in frame with components of the apoptotic pathway allow conditional dimerization and apoptosis of the transduced cells after administration of a non-therapeutic small molecule dimerizer (Belshaw et al., 1996; Spencer et al., 1996; MacCorkle et al., 1998; Straathof et al., 2005). Examples include the inducible FAS (iFAS) or inducible Caspase9 (iCasp9)/AP1903 systems (Clackson et al., 1998; Di Stasi et al., 2011).

Genetic modification of cells with a protein expressed in the plasma membrane (Hewitt et al., 2007), allows cell removal after administration of a specific monoclonal antibody. For example, retroviral delivery of the CD20 molecule into T cells and anti-CD20 monoclonal antibody treatment post T cell infusion has been validated in preclinical models as a suicide gene strategy (Introna et al., 2000; Serafini et al., 2004; Griffioen et al., 2009). As an extension of this concept, other interesting pre-clinical models have been investigated: Kieback et al. introduced a 10 amino acid tag of c-myc protein into the TCR sequence allowing elimination after monoclonal antibody administration (Kieback et al., 2008), whereas a group from London generated a novel compact suicide gene (RQR8) combining epitopes from CD34 and CD20 enabling CD34 selection, cell tracking, as well as deletion after anti-CD20 monoclonal antibody administration (Philip et al., 2014), and finally, another approach has used truncated human EGFR polypeptide/anti-EGFR monoclonal antibody (Wang et al., 2011).

**Figure 1:**
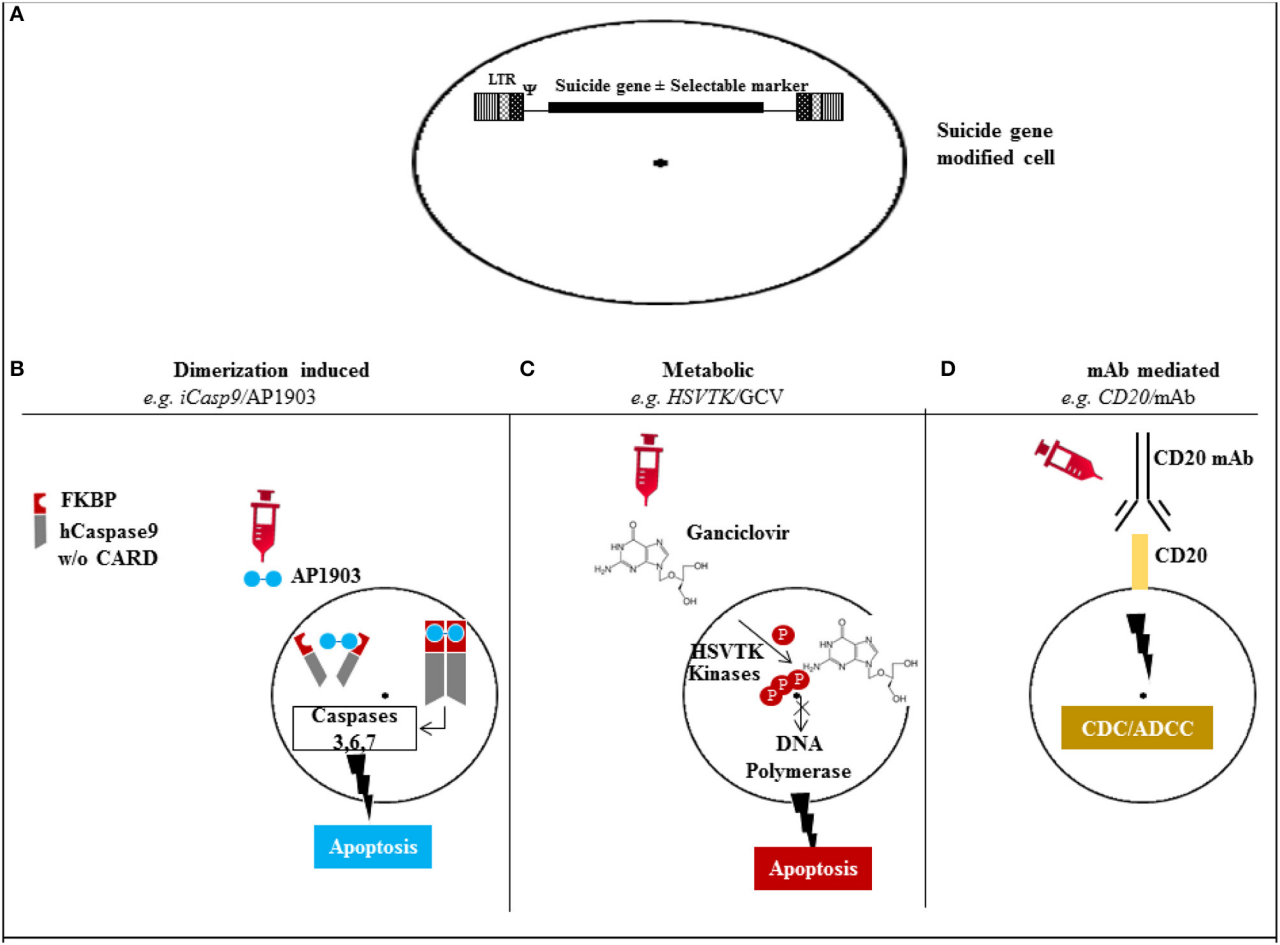
**Mechanism of action of the different suicide gene technologies. (A)** Suicide gene modification of cells of interest to allow conditional elimination in case of serious adverse events. Surface marker suicide genes, e.g., CD20, can also function as a selectable marker. **(B)** Dimerization induced e.g., iCasp9 protein with FKBP12-F36V binding domain joined to human caspase-9. Administration of AP1903 leads to dimerization of iCasp9 activating the intrinsic mitochondrial apoptotic pathway. **(C)** Metabolic, e.g., HSV/TK leads to phosphorylation of ganciclovir, and its triphosphate form (phosphorylated also through cellular kinases) incorporates into DNA with chain termination. **(D)** Monoclonal antibody (mAb) mediated, e.g., CD20 overexpression allows elimination after exposure to CD20 mAb through complement/antibody dependent cellular cytotoxicity (CDC/ADCC). *LTR: long terminal repeat, psi: retroviral packaging element, iCasp9: inducible Caspase9, CARD: Caspase recruitment domain, HSVTK: herpes simplex virus thymidine kinase, GCV: ganciclovir, mAb: monoclonal antibody*.

The mechanisms of action for the different suicide gene families is depicted in Figure 1, and characteristics of the major suicide gene systems investigated to date for adoptive immunotherapy are listed in Tables 1, 2.

**Table 1:** **Comparison of suicide genes for adoptive immunotherapy**.

**Category**	**Technology**	**Source**	**Activating agent**	**Mechanism(s)**	**References**
Metabolic	HSV-TK	Virus	GCV	1. Phosphorylated nucleotide disrupts DNA with cell death; 2. Apoptosis	Moolten, 1986; Bonini et al., 1997; Tiberghien et al., 2001; Ciceri et al., 2007, 2009; Vago et al., 2009; Oliveira et al., 2012
	CD	Bacteria, fungi	5-FC	Conversion of 5-FC to cytotoxic 5-FU	Tiraby et al., 1998
Dimerization inducing	iFAS	Human	Small molecule dimerizer	Dimerization and induction of apoptosis	Spencer et al., 1996
	iCasp9	Human	As above	As above	Spencer et al., 1993; Clackson et al., 1998; Di Stasi et al., 2011; Zhou et al., 2014
Therapeutic mAb mediated	CD20	Human	Anti-CD20 mAb	Complement dependent/antibody dependent cellular cytotoxicity	Introna et al., 2000; Serafini et al., 2004; Griffioen et al., 2009
	RQR8	Human	Anti-CD20	As above	Philip et al., 2014
	c-myc	Human	Anti-cmyc	As above	Kieback et al., 2008
	EGFR	Human	Anti-EGFR	As above	Wang et al., 2011

*Legend: HSV-TK, herpes simplex virus thymidine kinase; GCV, ganciclovir; CD, cytosine deaminase; 5-FC, 5-fluorocytosin; 5-FU, 5-fluorouracil; iFAS, inducible FAS; iCasp9, inducible Caspase9; mAb, monoclonal antibody; EGFR, Epidermal growth factor receptor*.

**Table 2:** ***Pros* and *cons* of suicide gene technologies**.

**Category**	**Example**	***Clinically validated***	***Pros***	***Cons***
Metabolic	HSV-TK	✓ (safe)	Gradual onset	Immunogenic in immunocompetent pts; unwanted elimination of modified T-cells with tx use of GCV
			Eliminates alloreactive cells	
Dimerization inducing	iCasp9	✓ (safe)	Rapid onset	Incomplete elimination, although of ≥90% of cells
			Eliminates alloreactive cells	
			Non Immunogenic	
			Use non-therapeutic agent	
Therapeutic mAb mediated	Surface molecule (*e.g*. CD20)	✘ (not validated)	Rapid onset	On-target toxicity from mAb
			Non immunogenic	
			No additional selectable marker required	

*Legend: HSV-TK, herpes simplex virus thymidine kinase; GCV, ganciclovir; iCasp9, inducible Caspase9; tx, therapeutic*.

The effectiveness of four suicide gene strategies has been compared *in vitro* using Epstein Barr virus cytotoxic T cells genetically modified to express HSV-TK, iCasp9, mutant human thymidylate kinase (mTMPK), or human CD20 codon optimized suicide gene. In this study, activation of HSV-TK, iCasp9, and CD20 ultimately resulted in equally effective destruction of transduced T cells. However, while iCasp9 and CD20 effected immediate cell-death induction, HSV-TK-expressing T cells required 3 days of exposure to ganciclovir to reach full effect, and mTMPK-transduced cells showed lower T-cell killing at all time-points (Marin et al., 2012).

Currently a one size fits all suicide gene is yet to be identified. The best suicide gene strategy should be designed for each specific application, taking into consideration the nature of target cells, the source of the suicide gene, the type of activating agent, the onset of action, and the elimination's kinetic of the target population.

Pre-clinical and clinical data employing the iCasp9 suicide gene system showed preferential elimination of cells with high iCasp9 transgene expression with sparing of quiescent cells. While this can represent an advantage when used for DLI, sparing viral and fungal reactive T cells (Zhou et al., 2014), complete elimination of CAR/TCR redirected T cells or HSCs may be necessary for the adverse event to abate. Therefore selection of cells with bright transgene expression, or combination of two suicide genes is advisable. Several strategies can be employed to ensure that all the “gene corrected” cells harbor the suicide gene. Cells may be transduced with a bicistronic vector with the suicide gene before an IRES or 2A sequence, or alternatively a selection strategy is used with introduction of a selectable marker.

The source of the suicide gene is also an important component of the strategy design. For example, in contrast to the iCasp9 suicide gene almost completely human derived, viral derived systems, such as HSV-TK, proved immunogenic in immune-competent patients with limited persistence of HSV-TK cells (Traversari et al., 2007).

Activating agents can also have varying effects, affecting the choice of which suicide gene system utilize for a given application. For example, AP1903 is a biologically inert small molecule dimerizer, whereas ganciclovir can also be used as a therapeutic agent to treat cytomegalovirus reactivation, precluding its use in patients receiving HSV-TK modified cells. The use of therapeutic monoclonal antibodies, in case of CD20 suicide gene or other surface markers, can lead to on-target effects. Therefore the adoption of molecules/antibodies with low toxicity profiles are preferred. Rapid onset of action of iCasp9 may be preferred to shut down a potentially fatal GVHD event. However a more gradual onset of action might be preferred to preserve a graft versus tumor effect.

Finally, resistance mechanisms can develop with these new technologies, and GCV-resistant truncated HSV-TK forms have been observed (Garin et al., 2001).

Currently, only HSV-TK/GCV and iCasp9/AP1903 have entered clinical trials to enhance the safety of cellular therapeutics for hematologic malignancies.

## Clinically validated suicide genes for control of GVHD

Two suicide genes have been validated in the clinic for control of GVHD from administration of donor T cells after HSCT in an effort to enhance immune recovery and maximize graft-vs.-leukemia (GVL) effect: the iCasp9 and the HSV-TK suicide genes. Clinical results are summarized in Table 3.

**Table 3:** **Larger studies of suicide gene modified donor lymphocyte infusion**.

**Graft**	**Disease**	**Suicide**	**Relapse N**	**N (aGVHD;**	**N (cGVHD;**	**N, Clinical**	**References**
	**status (N)**	**gene (N)**		**response)**	**response)**	**response**	
TCD-Haplo	CR (9) AD (1)	iCasp9 (10)	4	4 (CR)	0	5 CCR	Di Stasi et al., 2011; Zhou et al., 2014
N/A	Relapse or EBV-PTLD (8)	HSV-TK (8)	N/A	2 (CR)	1(PR)	3 CR, 2 PR	Bonini et al., 1997
TCD-MRD	AD (5) CR (3) CP (4)	HSV-TK (12)	2	5; CR 3/5	1 (CR)	4 (CR/CCR)	Tiberghien et al., 2001
MRD, MMRD	Relapse (30)	HSV-TK (23)	N/A	4 (CR)	1 (clinical benefit)	6 CR, 5 PR	Ciceri et al., 2007
TCD-Haplo	AD (20) CR (30)	HSV-TK (28)	17	10 (CR)	1(CR)	5 CR, 11 CCR	Ciceri et al., 2009

*Legend: GVHD, graft-versus-host-disease; a, acute; c, chronic; N, number; TCD, T cell depleted; haplo, 5/10 HLA matched haploidentical; (C)CR, (continuous) complete remission; AD, active disease; iCasp9, inducible Caspase9; D, day(s); EBV-PTLD, Epstein-Barr virus post-transplant lymphoproliferative disease; HSV-TK, herpes simplex virus thymidine kinase; NA, not available/applicable; PR, partial response; (M)MRD, (mis)matched related donor; CP, chronic phase*.

### HSV-TK suicide gene

Bonini et al. demonstrated the efficacy of HSV-TK suicide gene modified T cells in controlling GVHD in 8 patients who received DLI after HSCT for disease relapse or EBV post-transplant lymphoproliferative disease (PTLD). Importantly, a GVL effect attributed to the DLI was demonstrated in five patients (Bonini et al., 1997).

Subsequently, Tiberghien reported on 12 patients with hematologic malignancies who underwent HLA-matched related donor allo-HSCT (Tiberghien et al., 2001), and received HSV-TK DLI on the day of transplantation. Treatment with GCV alone resulted in complete remission (CR) in two of the three patients with aGVHD and CR was achieved with the addition of steroids in the third. GCV treatment also resulted in CR for the patient with cGVHD (Tiberghien et al., 2001).

The anti-tumor effects of HSV-TK DLI was studied in 23 patients with relapsed hematologic malignancies (Oliveira et al., 2012), where a clinical benefit was demonstrated in 65% of the cases. The development of antibodies against HSV-TK did not preclude a GVL effect, as the patients remained in complete remission thereafter, possibly due either to survival of a low T cell number in the periphery sufficient for immune surveillance, or abrogation of minimal residual disease. Eventually, the gradual elimination of HSV-TK cells can also contribute to a protracted GvL effect. GVHD was successfully controlled abrogated by the safety switch in this trial, as well (Traversari et al., 2007). Of note, infused T cells persisted *in vivo* up to 14 years after infusion (Oliveira et al., 2012). The largest study with 28 patients receiving HSV-TK engineered DLI after T cell depleted haplo-HSCT was published in 2009 (Ciceri et al., 2009). GVHD was successfully controlled with daily GCV administered for 2 weeks, with no cases of GCV resistance, progression from acute to chronic GVHD, and no GVHD-associated deaths. For patients with primary acute leukemia transplanted in remission the non-relapse mortality at 3 years was 19%. All patients in remission 3 years after transplant remained so in the following years (longest follow up 9 years) (Ciceri et al., 2009; Oliveira et al., 2012) Additional indirect evidence suggesting a GVL effect was the finding of *de novo* loss of mismatched HLA expression on leukemic blasts in one patient at the time of relapse (Vago et al., 2009).

### iCasp9 suicide gene

Spencer et al. (1993) and Clackson et al. (1998) demonstrated the ability to control signaling pathways through the administration of lipid permeable synthetic ligands, inducing conditional dimerization of intracellular proteins. They generated an inducible Casp9 suicide gene consisting of *FKBP12-F36V* domain linked, via a flexible *Ser-Gly-Gly-Gly-Ser* linker, to *ΔCaspase 9*, which is Caspase without its Caspase activator recruitment domain (Straathof et al., 2005). *FKBP12-F36V* consists of a *FKBP* domain with a substitution at residue 36 of phenylalanine for valine, binding synthetic dimeric ligands, such as AP1903 (Iuliucci et al., 2001), with high selectivity and subnanomolar affinity. The transgenic cassette was redesigned later to include a truncated CD19 (Δ*CD19*) molecule, serving as selectable marker to ensure ≥90% purity (Zhou et al., 1991; Fujimoto et al., 1998; Tey et al., 2007). After preclinical validation (Straathof et al., 2005; Tey et al., 2007), Brenner and collaborators reported their early results of a phase I clinical trial using the iCasp9 system (Di Stasi et al., 2011). Recipients of CD34-selected haplo-HSCT for hematological malignancies received escalating doses of iCasp9-modified allo-depleted (Amrolia et al., 2006; Tey et al., 2007) T cells from day 30 onwards. The iCasp9-modified T cells expanded and persisted for at least two years in surviving patients. In four patients who developed aGVHD the administration of 0.4 mg/kg AP1903 resulted in apoptosis of ≥90% of iCasp9-modified T cells within 30 min, followed by the rapid (within 24 h) and permanent abrogation of GVHD. Remarkably, residual iCasp9-modified T cells were able to re-expand, contained pathogen-specific precursors, without further GVHD. Although T cells recognizing tumor-associated antigens (TAAs) could be reactivated *ex vivo* from the peripheral blood before and after AP1903 infusion, three of the four patients receiving AP1903 had disease relapse, compared to only one of six patients who were not so treated, raising the concern that elimination of alloreactive cells would hamper GVL (Zhou et al., 2014), because of the potential co-expression of minor histocompatibility antigens on hematopoietic and non-hematopoietic tissues (de Bueger et al., 1992; Wang et al., 1995; Meadows et al., 1997; Vogt et al., 2000). The relative contribution of allo-reactive cells, as compared with TAAs specific T cells is not difficult to quantify, however since most TAAs are aberrantly expressed self-proteins resulting in T cells with low-affinity TCR, it is possible that the alloreactive component is more determinant for GvL. Additionally, although low frequency TAA specific T cells are transferred to patients after allo-HSCT or DLI, they do not persist (Rezvani et al., 2005, 2007), potentially due to activation-induced apoptosis (Molldrem et al., 2003), or terminally differentiated effector memory phenotype (Brenchley et al., 2003).

Given the successful abrogation of GVHD *in vivo*, several ongoing clinical trials have replaced the time consuming *in vitro* allo-depletion step with *in vivo* allo-depletion using AP1903 for those developing GVHD in the haploidentical (Clinicaltrials.gov identifier NCT01494103; NCT02065869; NCT01744223), or matched related settings, (Clinicaltrials.gov identifier NCT01875237).

In both the HSV-TK and iCasp9 studies, infusion of suicide gene modified cells aided non-gene modified T cell immune reconstitution (Ciceri et al., 2009; Bondanza et al., 2011; Di Stasi et al., 2011), as a consequence of interleukin-7 secretion by gene modified cells (Vago et al., 2012). The lack of further aGVHD in these studies might suggest either (i) complete elimination of allo-reactive cells, or (ii) development of peripheral tolerance. Additionally, the incidence of cGvHD was low in the HSV-TK T cell studies, and absent in the iCasp9 trial (Di Stasi et al., 2011; Zhou et al., 2014), and lymphocytes recovering from infused HSCs did not cause GVHD likely because of thymic education (Vago et al., 2012).

## Suicide genes application for the safety of genetically redirected T cells

TCR (Robbins et al., 2011) or CAR redirected T cells (Porter et al., 2011; Kochenderfer et al., 2012; Brentjens et al., 2013) have been successful implemented in several clinical trials. However adverse events including autoimmunity or off-target effects have been reported. Autoimmune phenomena manifested because the targeted antigens was shared also on normal tissues, (Yee et al., 2000; Dudley et al., 2002; Johnson et al., 2009; Parkhurst et al., 2011; Morgan et al., 2013) whereas off-target cardiac toxicity after high-avidity MAGE-A3 TCR T cells infusion was attributed to cross-reactivity with the Titin peptide in the striated muscles (Linette et al., 2013). Other toxicities observed included cytokine release syndrome (Brentjens et al., 2010; Kochenderfer et al., 2012), organ damage, including a case of fatal acute lung injury (Lamers et al., 2006, 2013; Morgan et al., 2010), and hypogammaglobulinemia from depletion of normal B cells (Kochenderfer et al., 2012). Although strategies including gamma globulin replacement, high dose corticosteroids, or the interleukin 6 receptor blocking antibody, tocilizumab exist for the management of hypogammaglobulinemia or cytokine release (Grupp et al., 2013; Davila et al., 2014), the use of a suicide gene is potentially able to irreversibly abrogate such toxicities. Pre-clinical experiments expressing the iCasp9 in conjunction with CAR CD19/CD20 T cells have proven the feasibility of such approach (Hoyos et al., 2010; Budde et al., 2013), and a phase 1 clinical trials in patients with sarcoma or neuroblastoma receiving iCasp9 T cells co-expressing a CAR against disialoganglioside GD2 molecule is ongoing (Clinicaltrials.gov identifier NCT01822652 and NCT01953900, respectively). If toxicity is related to the transduced T cells only, selectable markers could be obviated, especially in an autologous setting, provided that all the transduced cells also harbored the suicide gene in order to be eliminated in case of serious adverse events.

## Conclusions

Innovative technologies offer compelling opportunities for the optimization of gene therapy based approaches. Successful clinical validation of suicide gene strategies to control GVHD after allo-HSCT is now in advanced phase clinical studies, and introduction of suicide genes in conjunction with CAR modification of T cells for cancer immunotherapy is now undergoing phase 1 clinical testing.

Suicide genes have also been employed as cytotoxic strategy, *in vitro* and *in vivo* models (Huber et al., 1991; Clark et al., 1997; Nor et al., 2002; Nakayama et al., 2005; Hodish et al., 2009; Evans and Dey, 2011; Duarte et al., 2012; Mazor et al., 2012), including combination with replication competent oncolytic viruses (Ahn et al., 2009; Kaur et al., 2009), with some evidence of clinical benefit in solid tumors (Pandha et al., 1999; Freytag et al., 2003, 2007; Nemunaitis et al., 2003; Voges et al., 2003; Li et al., 2007; Xu et al., 2009).

Finally, non-integrating vectors (Banasik and McCray, 2010), strategies for the replacement or correction of defective genes (Narsinh et al., 2011; Mukherjee and Thrasher, 2013; Li et al., 2014), together with effective suicide gene strategies, may lead to a more broad application of stem cell or inducible pluripotent stem cell based applications in cancer and regenerative medicine.

## Author contributions

All the authors contributed to conception, acquisition, and analysis of data, participated in the manuscript draft preparation, revision and approved and revised the final version.

### Conflict of interest statement

The authors declare that the research was conducted in the absence of any commercial or financial relationships that could be construed as a potential conflict of interest.
